# Repeated competition influences neural response, but not neurogenesis or physiology, in the fighting fish *Betta splendens*

**DOI:** 10.1242/jeb.252766

**Published:** 2026-10-06

**Authors:** Jeremy A. Spool, Alexander J. A. Heywood, Ruth E. W. Berganross, Elaine A. Aquino, Temidayo D. Bambe, Kelly J. Wallace

**Affiliations:** ^1^ Department of Biology, Amherst College, Amherst, MA 01002, USA; ^2^ Department of Psychological and Brain Sciences, University of Massachusetts Amherst College of Natural Sciences, Amherst, MA 01003, USA

**Keywords:** Aggression, Territoriality, *Betta splendens*, pS6, Doublecortin, Cortisol

## Abstract

Territorial competition has costs such as wounds received, energy spent and elevated stress. How does *Betta splendens*, a species that has been artificially selected for enhanced territorial aggression, maintain high levels of metabolically costly displays (gill flaring) over repeated days of territorial competition? Here, we conducted two experiments. The first exposed male *B. splendens* to a size-matched unresolvable competition (a mirror) daily for 3 weeks. We found that *B. splendens* indeed maintained gill flaring throughout the competition schedule and exhibited no baseline stress impacts (measured via a scototaxis assay). We then exposed *B. splendens* to 1, 2 or 3 weeks of competition and quantified cortisol levels, neurogenesis and the neural response in brain regions associated with social processing. We again found that *B. splendens* showed consistently elevated gill flaring and no impact on stress (measured via waterborne cortisol). Competition-exposed fish displayed an increased neural response in the caudal supracommissural nucleus of the ventral telencephalon (Vs), a region that is homologous to the mammalian medial amygdala/bed nucleus of the stria terminalis and a central mediator of social decision making. The neural response in this same region positively correlated with individual variation in gill flaring. Competition did not influence neurogenesis (doublecortin expression). Lastly, a measure of reproductive investment (gonadosomatic index) did not correlate to behavior but did correlate to cortisol levels, suggesting a cohesive physiological state related to reproduction in this species. Taken together, this work details the mechanisms that are relevant to the maintenance of costly aggressive behavior.

## Teaser

**Highlighted Article:** *Betta splendens* maintain metabolically costly territorial aggression for weeks with no impacts on cortisol, neurogenesis or behavior; the neural response in the extended amygdala increases during competition and correlates to aggression.

## INTRODUCTION

Intraspecific competitions structure the social lives of many animals, determining access to territory (Eckert and Weatherhead, 1987; Elwood et al., 2006; Robertson, 1986), food (Armstrong, 1991; Ewald and Carpenter, 1978), mating opportunities (Austad, 1983; Clutton-Brock et al., 1979) and other crucial resources (Hsu et al., 2006). These competitions can take on many different forms. Biting (McGlone, 1985), grappling (Austad, 1983), displaying bright plumage (Pryke et al., 2002) or weaponry (Elwood et al., 2006), and singing (Krebs et al., 1981) are only a few examples of the many behaviors that can serve a competitive function. While success in these competitions can carry fitness benefits (Maynard Smith and Price, 1973), competition also comes with a number of costs, such as the risk of injury (Maan et al., 2001; McPeek and Crowley, 1987; Neat et al., 1998), the loss of time which could be spent on other activities (Abrahams et al., 2005; Briffa and Elwood, 2004), and exposure to predation (Brick, 1999; Jakobsson et al., 1995; Marler and Moore, 1988).

Winning or losing a competition can modulate future behavior. In several species of fish (Frey and Miller, 1972; Hsu and Wolf, 2001; McDonald et al., 1968), the copperhead snake *Agkistrodon contortrix* (Schuett, 1997) and the cricket *Gryllus bimaculatus* (Khazraïe and Campan, 1999), individuals that have previously lost an aggressive encounter are significantly less likely to initiate a subsequent contest, engage in fewer overtly aggressive acts (Franck and Ribowski, 1987; Frey and Miller, 1972; Ginsburg and Allee, 1942; Huhman et al., 2003; Schuett, 1997; Seward, 1946) and display more avoidant or submissive behaviors (Huhman et al., 2003; Schuett, 1997; Wallen and Wojciechowski-Metzlar, 1985).

In the present study we asked: what are the impacts of repeated territorial competition on behavioral, physiological and neural systems? In order to examine the cumulative effects of repeated competition, we conducted experiments in a species that has been artificially selected for increased territorial aggression. The Siamese fighting fish *Betta splendens* is anabantid freshwater fish in the gourami family (Osphronemidae), native to Southeast Asia (Forsatkar et al., 2017; Portugal, 2023). Observations in the wild note that males typically chase away intruding male competitors and even females once mating has occurred (Jaroensutasinee and Jaroensutasinee, 2000). While wild *B. splendens* engage in territorial aggression, domesticated *B. splendens* have undergone extensive artificial breeding for enhanced aggressive behavior and bright coloration (Portugal, 2023; Verbeek et al., 2007). Domesticated male *B. splendens* in lab environments also perform intense, competitive behaviors which are stereotyped and well described (Portugal, 2023; Simpson, 1968). They perform a gill-flaring display during competitions in which they flare out their opercula. Gill flaring is metabolically costly as it reduces aquatic oxygen intake, eventually leading one individual to stop flaring and surface to take up oxygen via their air-breathing organ (Alton et al., 2013). When wild *B. splendens* are exposed to each other in a small aquarium, one male typically retreats after an average of 10 min (Jaroensutasinee and Jaroensutasinee, 2001). But domesticated *B. splendens* confined to small aquaria exhibit physical combat for nearly an hour (though this can be reduced to 10 min when a large enough aquarium is provided) (Goldstein, 1975). These behavioral observations suggest that domesticated *B. splendens* are adapted to support high levels of territoriality, but the physiological and neural mechanisms mediating these responses are largely unexplored.

Unsurprisingly, one potential cost of repeated competition is an elevated level of chronic stress (Ramamoorthy and Cidlowski, 2016; Creel, 2001; Sapolsky, 1992). For this reason, many species have evolved ways to resolve direct conflict quickly or avoid it entirely (Maynard Smith and Price, 1973; Clutton-Brock and Albon, 1979). In the present study, we first exposed adult male *B. splendens* to 3 weeks of daily size-matched unresolvable competition (exposure to their mirror image) to determine how long they were able to sustain display behaviors and to ask whether competitive behavior changed over time. We expected to observe a decrease in gill flaring over the course of repeated competition as a result of the high metabolic costs of maintaining the display (Alton et al., 2013; Castro et al., 2006; Abrahams et al., 2005). Chronic social defeat stress and chronic unpredictable stress models have been established in other fish species (reviewed in Lai et al., 2023), but have yet to be validated in *B. splendens*. Thus, we additionally performed periodic light–dark scototaxis assays (Maximino et al., 2010) to quantify potential signatures of chronic stress. The scototaxis assay has been validated pharmacologically in a variety of fish models (Saaristo et al., 2019; Maximino et al., 2011; Lai et al., 2023). With regards to chronic stress, zebrafish exposed to 2 weeks of chronic unpredictable stress exhibited an increase in the time spent in the dark portion of the scototaxis arena (Chakravarty et al., 2013). An experiment in *B. splendens* specifically found that acute exposure to arsenic increased time spent in the dark portion of the scototaxis arena (Tudor et al., 2019). But the present experiment is the first to ask whether repeated territorial competition influences scototaxis responses in *B. splendens*.

We then conducted a second experiment to further probe the physiological systems that support sustained aggression. We exposed male *B. splendens* to repeated competition for 1, 2 or 3 weeks and measured waterborne cortisol. Prior literature in this species has reported that cortisol is elevated after fights (Ramos et al., 2021); thus, we expected to see elevated cortisol in fish exposed to repeated competition. Given that the presence of male competitors negatively impacts reproductive success in this species (Bronstein, 1982), in the present study we also hypothesized that exposure to repeated competition would alter reproductive investment as measured via gonadosomatic index (GSI, the ratio of gonad mass to body mass).

Territorial aggression involves high demand on neural circuits involved in social decision making. In fact, the social brain hypothesis posits that the cognitive load of social decisions has driven increased brain volume in social species including fish (Ma et al., 2025). We hypothesized that the neural activity supporting aggression would wane over 3 weeks of repeated competition. There is a network of brain regions that show deep homology across taxa and are implicated in mediating social behaviors including aggression, parental care and reproductive behavior (Newman, 1999; Goodson, 2005). Similarly, regions of the brain that process stimulus salience are members of the conserved dopaminergic reward pathway (Adinoff, 2004). These two networks share projections and function in a collection known as the social decision-making network (O'Connell and Hofmann, 2011). In the present study, we quantified activation of these neural systems in three regions of the social decision-making network: the putative teleost homologs of the extended amygdala, lateral septum and nucleus accumbens. In *B. splendens* specifically, Everett et al. (2025) observed that exposure to a conspecific induced an increased neural response in multiple regions with putative homology to mammalian regions involved in social decision making (O'Connell and Hofmann, 2011).

Lastly, the social environment influences neurogenesis across a wide variety of taxa including birds (Barnea and Pravosudov, 2011), frogs (Almli and Wilczynski, 2009) and fish (Dunlap et al., 2013; Sørensen et al., 2007). We hypothesized that neurogenesis would increase as a function of repeated territorial competition in order to support this costly aggressive behavior. Fish are a particularly exciting model to investigate neurogenesis as adult fish have the most widespread neurogenesis of any vertebrate studied to date (Ganz and Brand, 2016). More specifically, socially driven neurogenesis has been observed in telencephalic regions relevant to social communication in electric fish (Dunlap et al., 2013), and in other fish species, dominant and subordinate individuals differ in neurogenesis patterns (Sørensen et al., 2007).

## MATERIALS AND METHODS

### Animals

All fish were housed according to Amherst College Institutional Animal Care and Use Committee (IACUC) regulations, protocol #2024-2. The IACUC oversees compliance with the Guide for the Care and Use of Laboratory Animals, Eighth Edition, published by the National Research Council of the National Academies as well as the USDA Animal Welfare Regulations and PHS Policy on Humane Care and Use of Laboratory Animals. Amherst College operates under the Office of Laboratory Animal Welfare (OLAW) Assurance D16-00534 (A3925-01).

In experiment 1, 20 adult male blue veiltail *Betta splendens* Regan 1910 were purchased from Smithland Pet Center (Hadley, MA, USA) in March 2024. Fish were housed individually in clear 10 inch×6 inch×6 inch (∼24 cm×15 cm×15 cm) tanks with a 3 inch (∼76 mm) water height. Water used was tap water treated with API Stress Coat+ and left to dechlorinate for 24 h prior to use. For enrichment, fish were given three plastic aquarium plants and an Indian almond leaf. A 30% water change was performed every other day and fish tanks were replaced on a biweekly basis.

In experiment 2, a separate cohort of 35 adult male veiltail *B. splendens* were purchased from Petco (Holyoke, MA, USA) in October 2025. Individuals were housed in Aquaneering Zebrafish 2.8 l tanks (ZT280) with constantly recirculating biofiltered distilled water. Tanks received a constant gentle water flow from the sump and therefore water changes were not performed on individual tanks; rather, 10% of the water was replaced in the sump twice a week. Tanks were replaced on a biweekly basis for cleaning.

In both experiments, tanks were visually isolated from each other with barriers made of black corrugated plastic. The rooms housing the tanks were heated to keep an ambient room temperature of ∼25–27°C, a humidity of 35–50% and an average water temperature of ∼25–28°C. Fish were fed one pellet daily of TetraMin Tropical Flakes and housed on a 12 h:12 h light:dark cycle. Animals were sexed based on fin morphology (Miyagi et al., 2021).

### Behavior – experiment 1

For 3 weeks, fish were exposed to either their mirror image or no stimulus once a day from Monday to Thursday, followed by a scototaxis assay on Friday (Fig. 1A, ‘Behavioral experiment 1’). Fish assigned to the mirror treatment were exposed to 4 inch×6 inch (∼10 cm×15 cm) mirrors in their home tank, adhered in landscape orientation along the short edge of the tank for 10 min. We note that in Dupeyron and Wallace (2023), 10 min competitions between domesticated *B. splendens* daily over 4 days was sufficient for pairs to establish consistent winner–loser dyads, and in Clotfelter et al. (2006), gill flaring during a 10 min exposure to a female was sufficient to predict subsequent domesticated male reproductive investment (measured via bubble nest size).

**Fig. 1.**
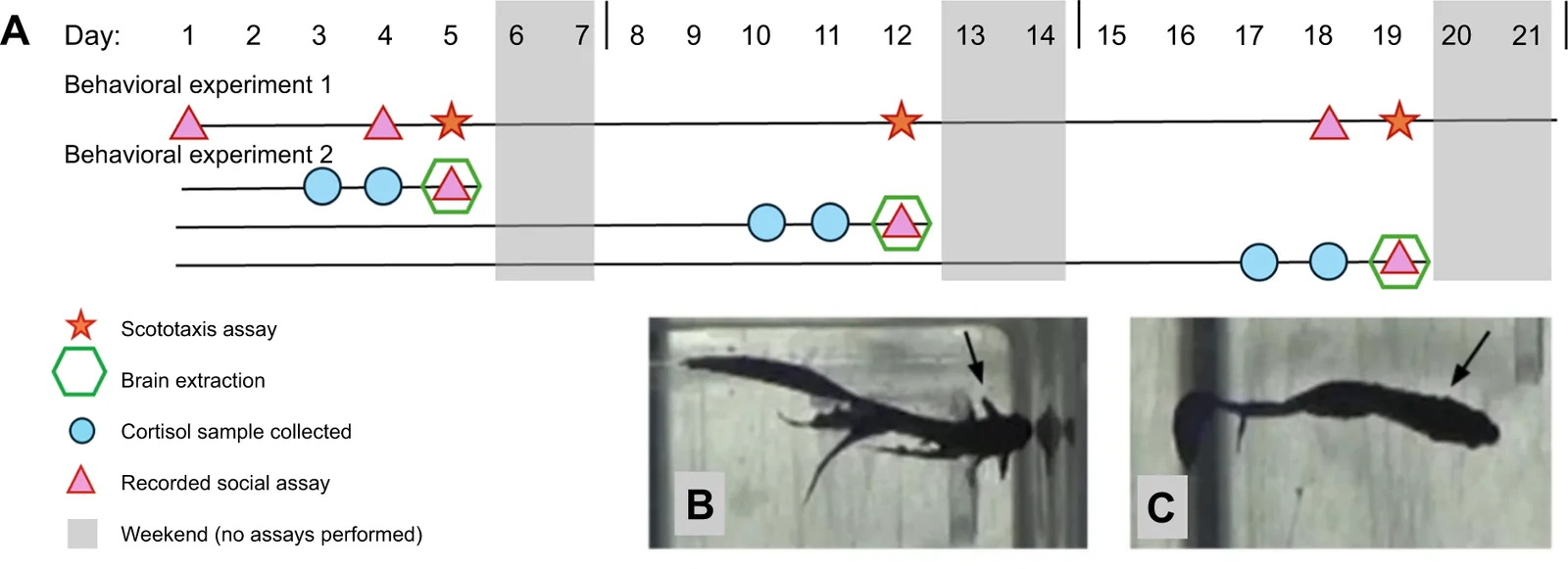
**Behavior experiments.** (A) An overview of the schedules of experiment 1 (all fish receiving 3 weeks of competition) and experiment 2 (fish receiving either 1, 2 or 3 weeks of competition), which differed in their time line and data collected. (B,C) Images of a fish gill flaring (B) and not gill flaring (C), with arrows identifying the opercula.

Note that during the trial, enrichment was removed from the tank to allow for overhead video recording, and then replaced after the end of the trial. The fish assigned to the control group received the same handling and then were recorded for 10 min with no additional stimulus presented. Videos of the competition assays were recorded with Sony HDRCX405 Handycams and analyzed for the proportion of the trial that each fish spent flaring its gills (henceforth referred to as ‘gill flaring’; Fig. 1B,C).

On Friday of each week, all fish were exposed to a 10 min scototaxis assay. Assays took place in 18 inch×6 inch×6 inch (∼46 cm×15 cm×15 cm) tanks filled to a depth of 2.5 inches (∼64 mm), which were lined on the walls and floor with black corrugated plastic on one half and white corrugated plastic on the other half. The water in each scototaxis tank was replaced between each assay. Overhead videos were analyzed for the proportion of time spent in the black half of the tank. More time spent in the black half of the tank, where the fish is better concealed, is considered a proxy for anxiety, whereas more time spent in the white half reflects a higher willingness to explore a ‘riskier’ area and therefore lower presumed anxiety (Maximino et al., 2010).

### Behavior – experiment 2

Fish were randomly assigned to a group to undergo repeated competitions for 1, 2 or 3 weeks (Fig. 1A, ‘Behavioral experiment 2’). Within each week, fish were randomly assigned to either be exposed to a mirror or be exposed to a white plastic card control. Fish were presented with a mirror or plastic card control in their home tank each week day between 10:00 h and 13:00 h for 10 min. Fish were fed at least 15 min prior to experiments. At the start of the assay, the stimulus was placed against the front wall of the tank. Fish were allowed to interact with the stimulus for 10 min, then the stimulus was removed. Video recordings of the assay were not taken in order to minimize disturbance to the fish except for the final 3 days of the assay prior to euthanasia, described below.

The assay design was slightly different on the final 3 days of the experiment to allow for filming and to create a schedule conducive to assessing the neural response. For these final 3 days, the mirror assays lasted 15 min. Home tanks were moved from the aquaneering rack to a stainless steel table and placed on top of a white acrylonitrile butadiene styrene (ABS) plastic surface (Kinlink). White plastic sidings of the same material were cut to the dimensions of the tank and inserted on all sides of the tank to prevent fish from interacting with their reflections in the new environment. Fish were then habituated for 15 min. Following the habituation, a mirror or plastic card control was introduced on one end of the tank and behavior was recorded from overhead for 15 min using Sony HDRCX405 Handycams.

Behavioral videos from the three recorded trials of each fish were analyzed using the event-logging software CowLog (version 3.0.2) (Hänninen and Pastell, 2009). All trials were scored for gill flaring, location (in the near or far half of the tank relative to the mirror/control stimulus) and latency to first bite attempt. Fish were considered to be flaring when their gills exceeded a 45 deg angle from their body. For location scoring, fish were considered to have crossed into the other half of the tank when their head was fully over the halfway point of the tank, which was marked on a transparent sheet placed on top of the video during scoring. The latency to first bite attempt and proportion of gill flaring prior to first bite attempt were recorded as measurements of escalation to physical combat.

A subset of fish (*n*=5) assigned to the 1-week group were run later than the rest of the 1-week cohort, during the final experimental week (i.e. when fish assigned to the 3-week group were being collected). We found no significant differences between the original 1-week group and this late-added 1-week cohort in the proportion of time spent gill flaring or the proportion of time spent near the stimulus; thus, they were combined for subsequent analysis.

### Waterborne hormone collection and ELISA quantification (experiment 2)

Three and two days prior to the end of the experiment, following behavioral trials, fish were immediately transferred to a plastic cup containing 200 ml treated water, covered with a towel to ensure a dark environment, and left undisturbed for 90 min. At 90 min, fish were transferred back to their home tanks. The water sample was transferred to a Nalgene wide-mouth LDPE bottle and frozen at −20°C.

To perform hormone extraction, the undiluted water samples were first thawed and then filtered using Fisherbrand Q5 filter paper (09-790-2F). The samples were extracted using a custom 3D-printed vacuum manifold attached to a vacuum that allowed for a flow rate of 10 ml min^−1^. The water sample was passed through a Sep-Pack Plus C18 cartridge (50818645) that was first equilibrated using 4 ml of methanol then activated with 4 ml of MilliQ water. After the sample was passed through the cartridge, the cartridge was flushed with 4 ml of MilliQ water. The cartridge was the wrapped in Parafilm and frozen at −20°C overnight. The following day, cartridges were reattached to the vacuum manifold and eluted using 4 ml of ethyl acetate into borosilicate glass tubes. Samples were then evaporated for 3–4 h under a constant stream of nitrogen gas at a flow rate where the water is barely being disturbed but not splashed. Samples were wrapped in Parafilm and stored at −20°C overnight. The following day, samples were reconstituted at room temperature using 10 μl of 100% ethanol and 190 μl of ELISA buffer. Samples were then processed for ELISA using a Cayman Cortisol Kit (500360) according to the manufacturer’s instructions. Samples were processed in triplicate. Any measurement with an intra-assay coefficient of variation (CV) larger than 20% was excluded from the data (*n*=9 out of 44) based on the manufacturer’s recommendation.

### Neural tissue collection (experiment 2)

To assess the neural response to social contexts, we labeled telencephalic neural tissue for phosphorylated ribosomal protein S6 (pS6). pS6 identifies ribosomal proteins that have been phosphorylated in roughly the previous hour, corresponding to an increase in translation (Butler et al., 2018; Magnuson et al., 2012; Dupeyron and Wallace, 2023). To assess neurogenesis in response to repeated competition, we labeled telencephalic neural tissue for doublecortin (DCX) expression. DCX is typically expressed in the early neuronal differentiation stage (Ganz and Brand, 2016) and is transiently expressed by newly generated neurons in the telencephalon in fish (Tozzini et al., 2012).

On the final day of the experiment following behavioral trials, fish tanks were covered with a towel for 90 min and left undisturbed. This 90 min time frame reflects prior literature demonstrating that pS6 expression peaks around 70–100 min (Corrales Parada et al., 2024; Everett et al., 2025). At 90 min, fish were humanely and rapidly euthanized by immersing them in an ice water bath. Final mass was collected and then fish were decapitated, and the heads were fixed in 4% paraformaldehyde for 4 h at 4°C. At this time, the testes were dissected out of the body cavity and weighed to determine a GSI score, which was calculated as mg testes mg^−1^ total body mass. Following fixation, the brain was extracted from the skull and immersed in 30% sucrose in 0.1 mol l^−1^ phosphate-buffered saline (PBS) overnight until the brain sank. Brains were frozen at – 80°C in Tissue-Tek OCT (25608-930) prior to cryosectioning into three identical series at 20 μm at −23°C onto Trubond 380 glass slides (50-340-33). Slides were stored at −80°C until processed for immunohistochemistry.

### Immunohistochemistry (experiment 2)

Slides were defrosted for 10 min at room temperature, after which a hydrophobic barrier was drawn around the edge using an ImmEdge Hydrophobic Barrier Pen (NC9545623). Tissue was rinsed in 0.1 mol l^−1^ PBS 5 times for 5 min each, and then blocked in 0.3% Triton X-100 (BioXtra, T9284), 0.1% Tween 20 (Fisher Scientific, BP337) and 10% Normal Donkey Serum (NDS, Jackson ImmunoResearch 017-000-121) in 0.1 mol l^−1^ PBS for 1 h. Slides were then transferred to primary antibody solution containing 0.3% Triton X-100, 0.1% Tween 20 and 5% NDS in 0.1 mol l^−1^ PBS, with primary antibodies targeting pS6 (mouse anti-pS6, 1:500, Cell Signaling Technology E2R1O) and DCX (rabbit anti-DCX, 1:250, Invitrogen 48-1200). Sections were incubated in primary antibody solution overnight at 4°C. The next day, slides were rinsed twice in 0.1 mol l^−1^ PBS for 30 min, and transferred to secondary antibody solution containing 0.3% Triton X-100, 5% NDS and secondary antibody (donkey anti-mouse 488, 1:1000, Invitrogen A21202; donkey anti-rabbit 594, 1:1000, Invitrogen A21207). Slides were incubated in the secondary antibody solution for 2 h at room temperature in the dark. Slides were finally rinsed in 0.1 mol l^−1^ PBS twice for 10 min and then coverslipped using Invitrogen ProLong Gold Antifade Mountant with DNA Stain 4′,6-diamidino-2-phenylindole (DAPI; Invitrogen, P36931) and cured overnight prior to imaging.

To help identify morphological landmarks for cell counting, the second series of tissue for each animal was stained for the neuronal marker HuC/D (Maruska et al., 2012) (mouse anti-HuC/D, 1:200, Invitrogen 16A11; followed by 1:1000 donkey anti-mouse 488 secondary, Invitrogen A21202) and tyrosine hydroxylase (rabbit anti-TH, 1:500, Millipore AB152MI; followed by 1:1000 donkey anti-rabbit 594, Invitrogen A21207) following the protocol described previously.

### Microscopy and cell quantification (experiment 2)

Slides were imaged at the Amherst College Biology Imaging Core on a Zeiss LSM710-Confocor3 confocal microscope at high resolution using 405 nm, 488 nm and 561 nm lasers for DAPI (nuclear stain), Alexa Fluor 488 (pS6) and Alexa Fluor 594 (DCX). A Zeiss Plan Apochromat 20×/0.8 NA objective was used for all imaging and the pinhole was adjusted to 1 AU on the longest wavelength.

Prior to the identification of specific brain regions, each tissue section was categorized for rostral–caudal position using the Magalhães Horn and Rasia-Filho *Betta splendens* telencephalic atlas (Magalhães Horn and Rasia-Filho, 2018), using the DAPI nuclear stain, as the DAPI morphology closely mirrored the Nissl stain from the Magalhães Horn and Rasia-Filho atlas. Images were categorized as atlas category E (−180 μm), F (−130 μm), G (−20 μm), H (0 μm) or I (+70 μm) (Fig. 2) and then labeled as broadly rostral or caudal.

**Fig. 2.**
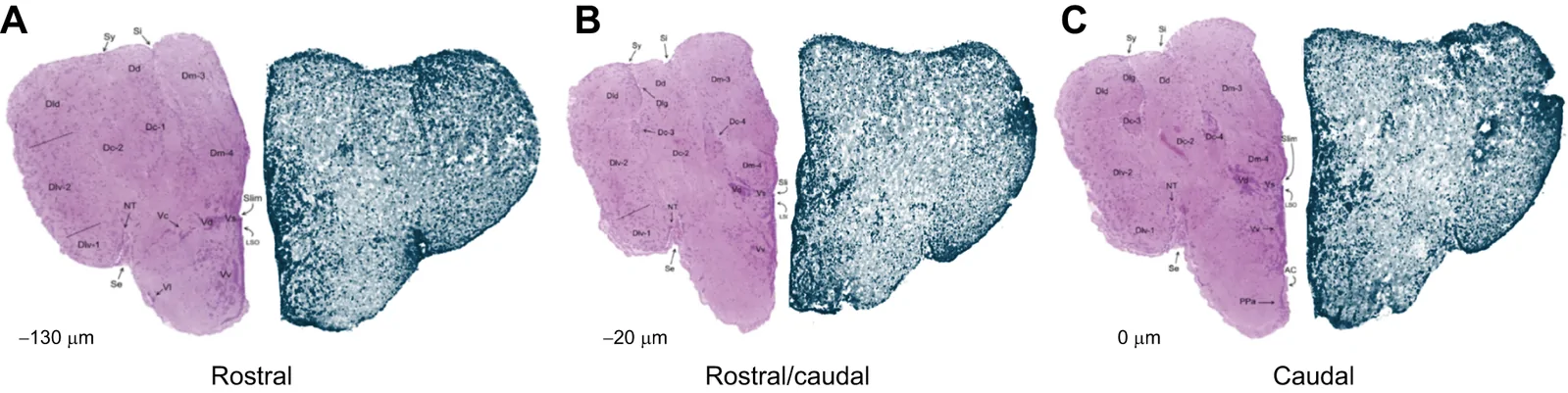
**Categorization of imaged telencephalic tissue from *Betta splendens*.** Tissue was categorized as rostral, rostral/caudal or caudal based on landmarks visible in the Magalhães Horn and Rasia-Filho atlas (sections F, G and H, respectively): in each case, the left image shows hematoxylin–eosin staining adapted from Magalhães Horn and Rasia-Filho (2018) and the right image shows DAPI staining from tissue processed immunohistochemically (present study). AC, anterior commissure; Dc, central zone of the dorsal telencephalon; Dd, dorsal zone of the dorsal telencephalon; Dl, lateral zone of the dorsal telencephalon; Dm, medial zone of the dorsal telencephalon; LSO, lateral septal organ; NT, nucleus taenia; PP, parvocellular preoptic nucleus; Se, sulcus externus; Si, sulcus inominatus; Slim, sulcus limitans; Sy, sulcus ypsiloniformis; Vc, central nucleus of the ventral telencephalon; Vd, dorsal nucleus of the ventral telencephalon; Vl, lateral nucleus of the ventral telencephalon; Vs, supracommissural nucleus of the ventral telencephalon; Vv, ventral nucleus of the ventral telencephalon.

While the Magalhães Horn and Rasia-Filho telencephalic atlas (Magalhães Horn and Rasia-Filho, 2018) provides an excellent general idea for region location, we additionally relied on landmarks determined by evaluating the information from the atlas, DAPI staining, HuC/D neuronal staining and tyrosine hydroxylase staining. Notably, in the DAPI channel, the distinction between the F/G/H atlas categorizations can be determined by viewing whether the dense DAPI labeling of the supracommissural nucleus of the ventral telencephalon (Vs)/dorsal nucleus of the ventral telencephalon (Vd) seems to ‘curve’ downward (F, −130 μm) or upward (G/H, −20 μm–0 μm) (Figs 2 and 3A). Unfortunately, we could not get a clear representation from our dataset of the preoptic area (POA). The POA exists at the ventral edge of the tissue and, because of challenges with tissue folding and shredding along the edges, our sample size was too small to quantify cell counts.

**Fig. 3.**
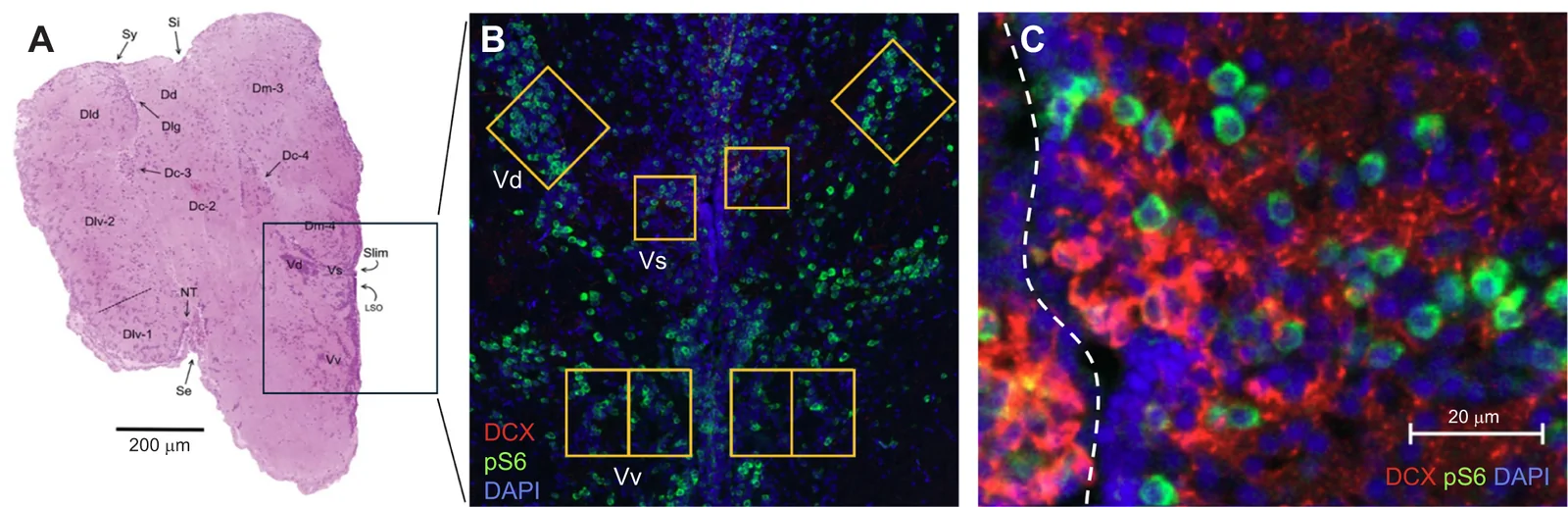
**Telencephalic tissue labeled for both a neurogenesis and neural response marker.** (A) The telencephalic atlas, adapted from Magalhães Horn and Rasia-Filho (2018). (B) Example ROI placement for all regions quantified for neurogenesis and neural response in the present experiment. Note that for all fish, the ROI placement was in the identical location for both the phosphorylated ribosomal protein S6 (pS6) and doublecortin (DCX) channels. (C) Example image near the Vs showing DCX labeling of cell bodies along the midline (dashed white line) and cellular processes more laterally. Note that the medial edge of the Vs ROI would be placed roughly where the scale bar is located in C.

All region of interest (ROI) placement and cell counting was conducted through Fiji (Image J 1.54p, Java 21.0.7, 64-bit). The ROI size (in micrometers) for each region was as follows: Vs: 50×50, Vd:70×70, ventral nucleus of the ventral telencephalon (Vv): 70×50 (Fig. 3B). For the Vv and Vs, the ROI was placed to not include cells in the midline itself as these cells appear to be distinct from interior tissues, and furthermore tissue quality on section edges is frequently folded or inconsistent. When necessary, the ROI was rotated to best capture the population of interest (e.g. Fig. 3B). Lastly, the Vv was a wide enough ROI that we halved the ROI to separately count a medial and lateral subpopulation.

pS6+ cells with any portion of their labeled cell body within the ROI were counted. Within-region cell counting was always performed by the same author (R.E.W.B. or J.A.S., blind to treatment). For all regions, if multiple sequential brain sections contained the ROI then all relevant sections were counted and an average was taken across these sections (typically two or three sections). Additionally, each hemisphere was counted separately and averaged. Each image was counted twice by the same scorer (which including a second ROI placement), and an average between the first count and second count is reported. In cases where a large difference between the first and second count was observed, a third count was conducted. For all regions, if one hemispheric count differed by more than 50% between the first and second count, a third count was conducted just on that image and the second and third count were averaged instead. If the first and second count differed by more than 20% (Vs and Vd) or 40% (Vv) on two or more hemispheric counts, a third count was applied to all images of the relevant region for that subject and the second and third count were averaged instead. Because prior literature has identified differences in neural response in the rostral and caudal sections of regions of the social behavior network (Everett et al., 2025), we report the average counts of the rostral and caudal sections separately.

For DAPI and DCX, optical density (OD) was calculated using the program CellProfiler (Stirling et al., 2021). This was because while DCX labels cell bodies along the midline, in the ventral telencephalic areas quantified in the present experiment, DCX labels cellular processes in a more fibrous mass (Fig. 3C). Similarly, DAPI shows more cell bodies in a small area than was feasibly countable. Note that the ROI for OD quantification was the same exact placement as the other channel for pS6 quantification. In CellProfiler, we converted the relevant channel image to binary and calculated the percentage of white space in the total frame to get the proportion of the ROI that was positively labeled.

### Statistical analysis

Data were analyzed in R version 4.5.1 (https://www.r-project.org/). The cowlogdata R package (https://github.com/kellyjwallace/cowlogdata) was used to process the raw output from CowLog into a data frame containing the proportion of time fish spent performing behaviors of interest in each trial. *P*-values, effect sizes and statistical test used for all behavioral and physiological statistical comparisons (including non-significant results) are reported in Table S1. *P*-values, effect sizes and statistical tests used for all statistical comparisons involving neural response (pS6) data (including non-significant results) are reported in Table S2. To assess relationships between categorical predictors and continuous response variables, Shapiro–Wilk tests were performed using the shapiro_test function from the rstatix package (https://CRAN.R-project.org/package=rstatix) to assess whether the data were normally distributed. For normally distributed data, a two-tailed two-sample *t*-test (two categories) or an ANOVA (three or more categories) was performed; for non-normally distributed data, either a Wilcoxon rank-sum test (two categories) or a Kruskal–Wallis test (three or more categories) was performed using functions from base R. Effect sizes reported for ANOVA or Kruskal–Wallis tests are bias-corrected (Kruskal–Wallis) or classic (ANOVA) η^2^ (eta-squared) obtained via the rstatix package. A paired *t*- or Wilcoxon test was performed if assessing the same subjects across multiple time points. To assess relationships between continuous variables, linear models were conducted using the lm() function in base R, with test statistics, effect sizes, and *P*-values reported using the summ() function in the jtools package (https://CRAN.R-project.org/package=jtools; Long, 2024). In the case of a ranked-based continuous measurement, we used a rank-based regression for linear models using the R package Rfit (https://CRAN.R-project.org/package=Rfit; Kloke and McKean, 2012). Note that when evaluating data that included cortisol levels, a Gamma model was conducted using the glm() function in base R and a Cragg–Uhler Pseudo-*R*^2^ is reported. We calculated the intraclass correlation coefficient to assess the reliability of gill flaring (to quantify individual variation) using the ICC package in R (https://CRAN.R-project.org/package=ICC; Wolack et al., 2012).

For pS6 expression data, FDR correction for multiple comparisons was conducted to account for the seven comparisons (Vs/Vd/Vv rostral and caudal, and an additional comparison for Vv medial and lateral, M and L) using the p.adjust function in base R at *n*=7. Vs DCX was not multiple test corrected as this was the only region analyzed for DCX expression. To assess the neural response across multiple regions simultaneously, we conducted a principal component analysis using the R package pvclust (Suzuki and Shimodaira, 2006) with the rostral and caudal pS6+ cell counts for each region (Vs, Vd, VvM and VvL) included. Loadings reported are eigenvalues and PC scores were compared between mirror- and control-exposed fish via a *t*-test.

## RESULTS

### Experiment 1: competition did not wane over 3 weeks of mirror exposure

To assess whether domesticated *Betta splendens* change their behavior during repeated territorial competition, we exposed fish to 3 weeks of daily competition. On the first day of the experiment, mirror-exposed fish gill flared significantly more than the control-exposed fish (*P*=0.002, *t*=3.55, Cohen's *D*=1.61), and this difference remained through to the end of the experimental schedule on day 18 (*P*<0.001, *W*=99, Cohen's *D*=2.26) (Fig. 4A). Mirror fish did not change their gill flaring over the course of the experiment. In this first experiment, the relative amount of gill flaring on the first day (rank order) was predictive of relative gill flaring on the final day of competition (*P*=0.015, *F*_1,15_=34.21, *t*=3.06, *R*^2^=0.48; Fig. 4B). There were low amounts of gill flaring on the first day by the control fish (presumably due to seeing their reflection on the side walls of the tank, which was fixed in experiment 2 using non-reflective inserts) but this significantly decreased and we observed almost no gill flaring on the final day (*P*=0.016, *V*=42, Cohen's *D*=1.07; Fig. 4A).

**Fig. 4.**
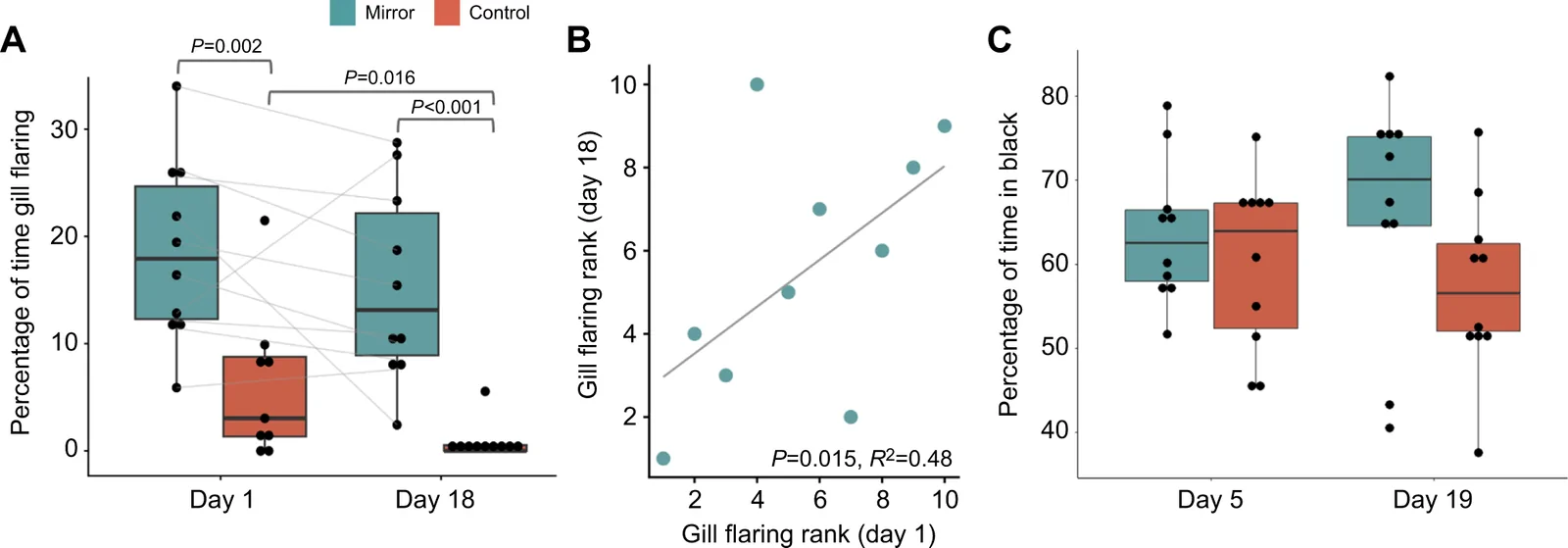
**Territorial aggression did not wane over 18 days or induce anxiety-like behavior.** (A) Experiment 1 consisted of fish exposed once a day to either a mirror (teal, *n*=10) or control (coral, *n*=10) stimulus for a total of 3 weeks. Mirror-exposed fish exhibited significantly more gill flaring than control-exposed fish on the first (*t*-test *P*=0.002) and final (Wilcoxon, *P*≤0.001) day of repeated competition. Mirror-exposed fish did not exhibit significant changes in aggressive behavior over the course of this extended competition. (B) Relative (rank) gill flaring to the mirror on day 1 significantly predicted relative gill flaring on day 18 (linear regression *P*=0.015); see lines connecting individuals in A. (C) Fish did not exhibit significant changes in anxiety-like behavior over the course of this extended competition. Boxplot middle lines represent the median value, box edges represent the first and third quartiles, and whiskers extend to data points within the 1.5 interquartile range of the box edge.

### Experiment 1: repeated competition did not influence scototaxis behavior

To assess whether repeated exposure to competition influenced anxiety-related behavior, we compared time spent in the black half of a scototaxis tank towards the beginning (day 5) and at the end (day 19) of the experimental schedule. Mirror-exposed and control-exposed fish did not differ in their scototaxis behavior on either day (Fig. 4C). Neither mirror-exposed fish nor control-exposed fish changed their scototaxis behavior between the start and end of the experimental schedule (Fig. 4C). At an individual level, aggressive behavior did not predict behavior in a scototaxis assay the following day. This pattern was true at both the start and end of the experimental schedule (Fig. S1A,B). When combining all individuals, scototaxis behavior on day 5 did not significantly predict scototaxis behavior on day 19, but overall the amount of time spent on the black side of the scototaxis tank (61%) was consistent with prior literature on this species (Dupeyron and Wallace, 2023).

### Experiment 2: gill flaring was comparable across the two experiments

To understand the physiological and neural consequences of sustained aggression, we conducted a second experiment in a separate cohort of fish and collected tissue at three different time points. To validate that the two experiments reflected the same broad social paradigm, we compared the amount of gill flaring when exposed to a mirror between the two experiments. While not a direct comparison, as experiment 1 averaged across 3 days each separated by a week whereas experiment 2 averaged across 3 sequential days, the mean (±s.d.) proportion of time spent gill flaring in experiment 1 (18±8%) and experiment 2 (13±9%) did not significantly differ from each other.

### Experiment 2: fish showed aggressive behavior towards the mirror but not the control stimulus

We quantified territorial behavior across 3 consecutive days. We observed consistent individual variation in gill flaring with high reliability across the 3 days (ICC=0.829). When individuals were ranked from lowest to highest average gill flaring over the final 3 days, this consistency could be visually observed: fish showed highly similar values across each of their three gill flaring scores (Fig. 5A). Once one individual's gill flaring data were removed as an outlier as it was over 60% higher than all other values recorded, the amount of gill flaring observed on the first of the 3 days consistently predicted the amount of gill flaring on both the second (*P*<0.001, *F*_1,15_=34.21, *R*^2^=0.67) and third day (*P*<0.001, *F*_1,15_=33.00, *R*^2^=0.67). Thus, for all additional analyses, we report the average value of gill flaring behavior across the 3 days.

**Fig. 5.**
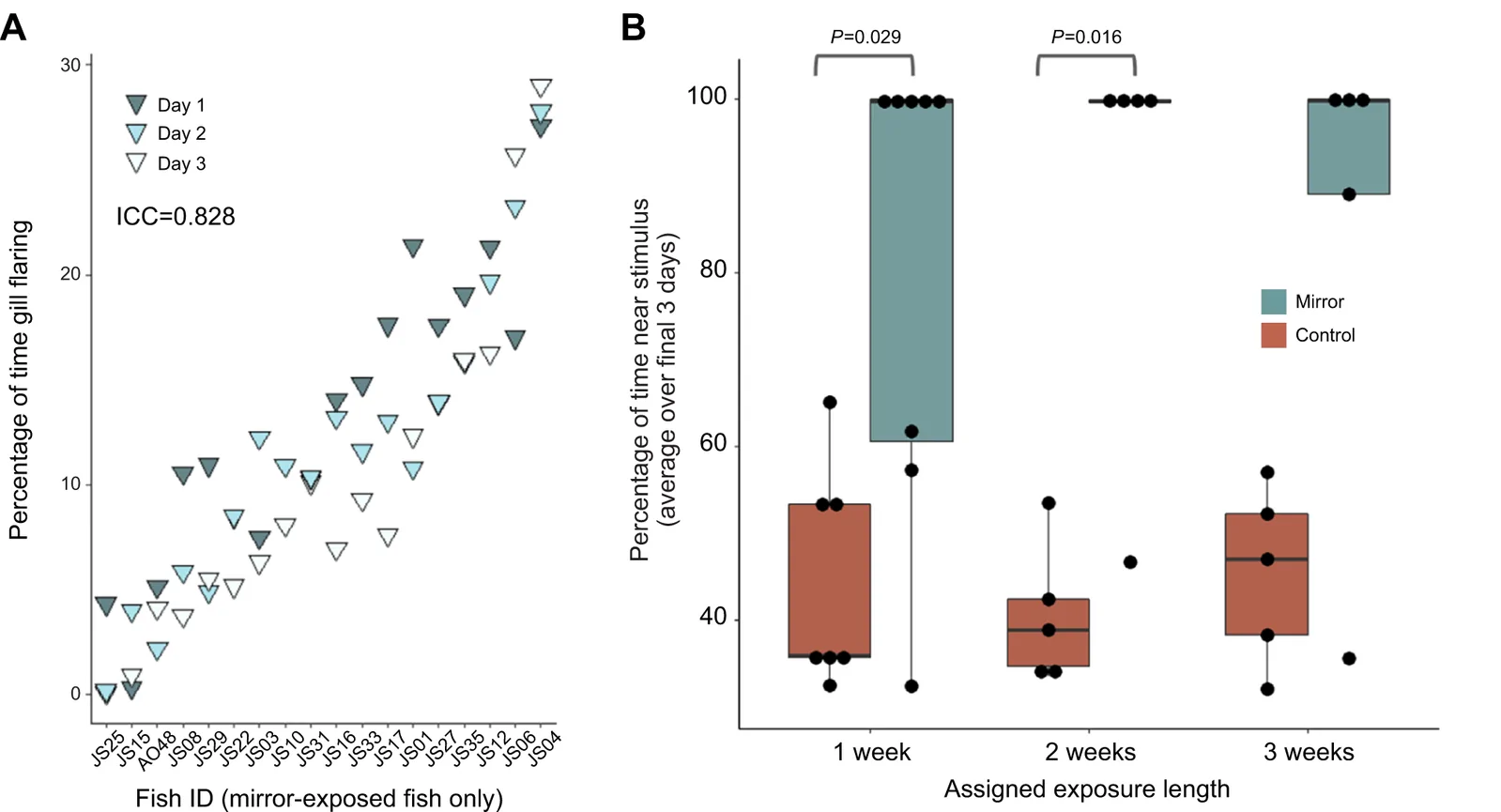
**Territorial aggression was consistent but did not wane when assessed over multiple time points.** (A) Experiment 2 consisted of fish exposed once a day to either a mirror or a control stimulus for 1 (*n*=8/7 mirror/control), 2 (*n*=5/5 mirror/control) or 3 weeks (*n*=5/5 mirror/control). We averaged gill flaring behavior across the final 3 days of exposure, which was appropriate because we observed high consistent individual variation in gill-flaring responses to the mirror (ICC=0.829). (B) Mirror-exposed fish spent more time near the stimulus than control-exposed fish (all weeks combined Wilcoxon, *P*<0.001; week 1 Wilcoxon, *P*=0.029; week 2 Wilcoxon, *P*=0.016; week 3, Wilcoxon *P*=0.09). Boxplot middle lines represent the median value, box edges represent the first and third quartiles, and whiskers extend to data points within the 1.5 interquartile range of the box edge.

Combining all exposure lengths, fish exposed to the mirror spent on average 84.5±25% (mean±s.d.) of each trial on the half of the tank nearer the stimulus, which was significantly more than the control fish (43.3±10%; *P*<0.001, *W*=37, Cohen's *D*=−2.12). This significant difference was observed within the first 2 weeks but was a non-significant trend on the third week (week 1: *P*=0.029, *W*=9, Cohen's *D*=−1.71, week 2: *P*=0.016, *W*=1, Cohen's *D*=−2.74, week 3: *P*=0.09; Fig. 5B).

Control fish were expected to show no preference for either half of the tank, but the average proportion of time control fish spent near the control stimulus (43.3%) was significantly lower than 50% (two-tailed one-sample Wilcoxon test at μ=0.5, *P*=0.031, *V*=31, Cohen's *D*=−0.62), suggesting a slight aversion to the water disturbance caused by the stimulus placement. Unsurprisingly, fish exposed to the mirror spent more time gill flaring than fish exposed to the control stimulus, though we did not statistically test this as control fish universally did not flare. The average latency to first bite attempt for fish facing a mirror was relatively long (250±151 s, mean±s.d.; Fig. S2B), but the average latency to gill flare at the mirror was very short (7±7 s; Fig. S2C). No control fish made a bite attempt. The proportion of time a fish spent flaring before its first bite attempt was compared with the latency of that first bite attempt to determine whether a correlation existed between initial display vigor (represented by pre-bite flaring; Fig. S2D) and escalation to physical attacks (represented by latency to first bite; Fig. S2B), but no significant relationship was found between pre-bite flaring and bite latency.

### Experiment 2: behavior did not change over 3 weeks of repeated competition

Similar to experiment 1, there was no significant difference between 1 week, 2 weeks or 3 weeks of exposure to the mirror in the proportion of time spent flaring, latency to gill flare, latency to first bite attempt or gill flaring prior to the first bite (Fig. S2). Neither mirror-exposed nor control-exposed fish showed differences in time spent near the stimulus between 1, 2 or 3 weeks (Fig. 5B).

### Experiment 2: neural response in the extended amygdala differed between mirror-exposed and control fish

Of the brain regions in which we quantified pS6+ cells (Vs, Vd, Vv, the teleost putative homologs to the extended amygdala, nucleus accumbens and lateral septum), no regions or subregions exhibited significant differences between fish exposed to the mirror for 1, 2 or 3 weeks (caudal Vs results shown in Fig. S3A), or between fish exposed to the control stimulus for 1, 2 or 3 weeks. Thus, we combined all weeks to compare all mirror-exposed fish with all control-exposed fish. In doing so, we observed a significant difference between effect of exposure type on the mean count of pS6+ cells in the caudal Vs (*P*=0.041 after FDR correction, *t*=−3.029, Cohen's *D*=−0.55) (Fig. 6A), in which mirror-exposed fish had more pS6+ cells in the caudal Vs than control-exposed fish. This was not driven by overall cellular morphology, as the optical density of DAPI in the same ROI did not differ between mirror-exposed and control-exposed fish. The number of pS6+ cells did not correlate to the amount of gill flaring on the final day for fish exposed to the mirror, GSI or standard length in any region. When analyzing the neural response across multiple regions using a principal component analysis, we found that PC2 scores significantly differed between mirror-exposed and control-exposed fish (*P*=0.008; Fig. 6B). PC2 explained 15% of the data and loaded most strongly by rostral (+0.684) and caudal (+0.519) Vs pS6 expression (Fig. 6B). Both the medial (−0.302) and lateral (−0.348) regions of the caudal Vv loaded in the opposite direction to the Vs in PC2. A *post hoc* analysis of the caudal Vs (where we had observed the significant treatment effect) showed that the average amount of gill flaring over the final 3 days positively correlated with the neural response (*P*=0.015, *F*_1,12_=8.00, *R*^2^=0.35; Fig. 6C).

**Fig. 6.**
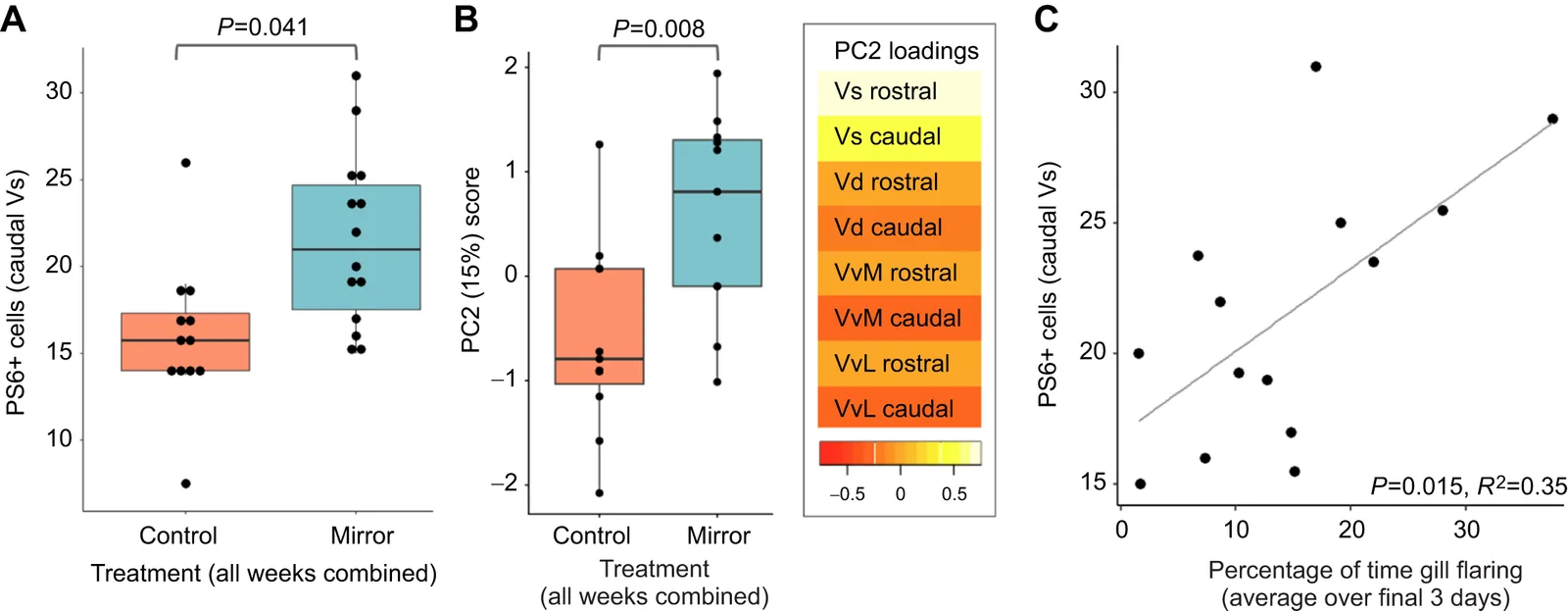
**Repeated territorial aggression of any length increased the neural response in the teleost homolog of the extended amygdala.** (A) In experiment 2, the caudal Vs neural response significantly differed between fish exposed to a mirror (*n*=14) or a control stimulus (*n*=12) (*t*-test *P*=0.041). (B) A principal component analysis found that PC2, which loaded most strongly by Vs neural response (inset), significantly differed between mirror-exposed and control-exposed fish. (C) Vs pS6 expression positively correlated to the amount of time spent in aggressive behavior towards a mirror in the final 3 days of the task (*n*=14) (linear regression *P*=0.015). Boxplot middle lines represent the median, box edges represent the first and third quartiles, and whiskers extend to data points within the 1.5 interquartile range of the box edge.

### Experiment 2: neurogenesis in the Vs did not differ between mirror-exposed and control-exposed fish

As shown in Fig. 3C, we were able to assess neurogenesis via DCX optical density in the rostral and caudal Vs. In the mirror-exposed fish, rostral and caudal DCX optical density did not differ between 1, 2 or 3 weeks of exposure. Combining all weeks, we then tested for a general exposure effect but found that DCX optical density in the rostral and caudal Vs did not differ between mirror-exposed and control-exposed fish (Fig. S3B,C).

### Experiment 2: repeated exposure to competition did not influence baseline cortisol

Fish exposed to a mirror competitor for 1, 2 or 3 weeks did not differ in their waterborne cortisol levels (Fig. 7A). In fact, when combining across weeks, mirror-exposed fish did not differ in cortisol levels from control-exposed fish (Fig. 7B). At an individual level, cortisol measurements did not correlate to the amount of gill flaring during the mirror exposure that same day.

**Fig. 7.**
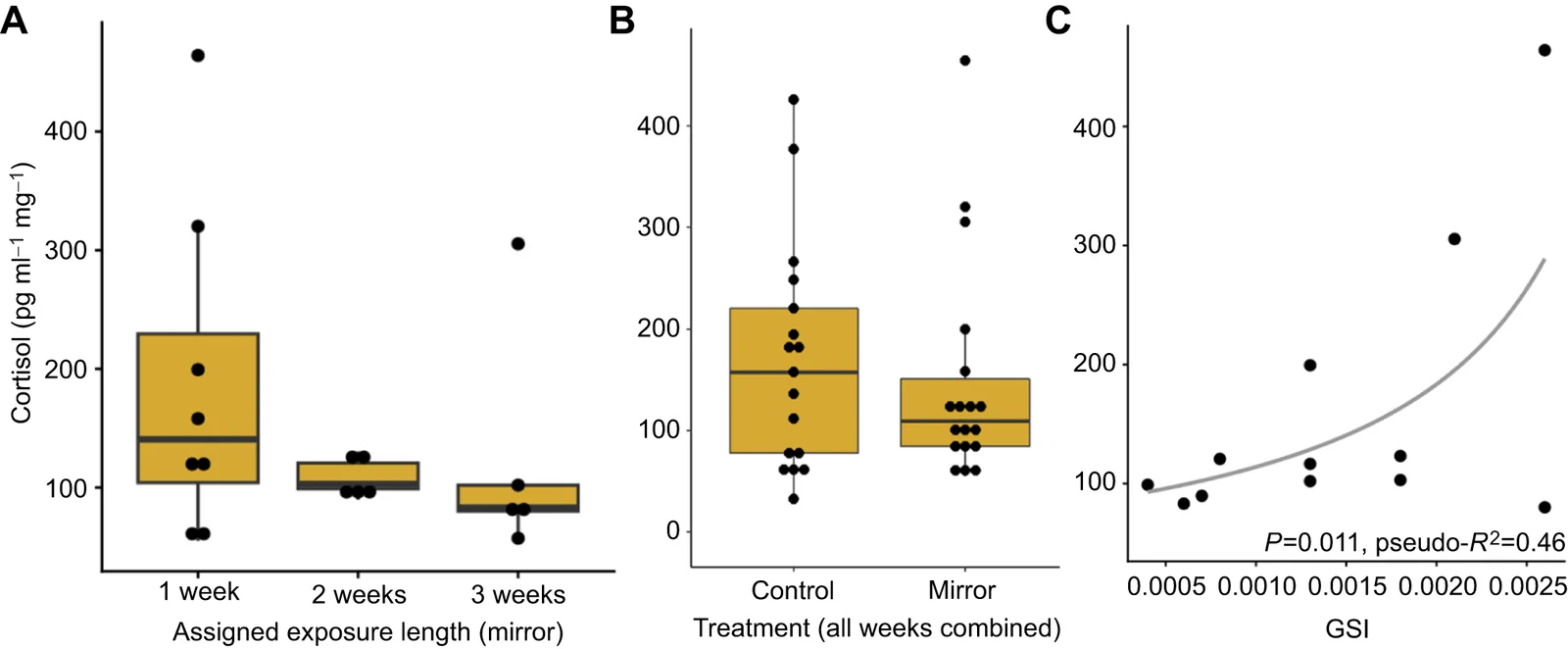
**Baseline cortisol levels did not reflect repeated competition but did correlate to gonadosomatic index.** (A) In experiment 2, fish assigned to 1 week (*n*=8), 2 weeks (*n*=5) or 3 weeks (*n*=5) of competition via exposure to a mirror did not differ in waterborne cortisol levels. (B) Combining fish from all assigned exposure lengths, waterborne cortisol levels did not differ between mirror-exposed (*n*=18) and control-exposed (*n*=17) fish. (C) Waterborne cortisol levels positively correlated to gonadosomatic index (GSI) in mirror-exposed fish (*n*=12) (generalized linear model *P*=0.011). Boxplot middle lines represent the median value, box edges represent the first and third quartiles, and whiskers extend to data points within the 1.5 interquartile range of the box edge.

### Experiment 2: reproductive investment did not predict mirror aggression but did correlate to baseline cortisol

Of the 18 fish exposed to the mirror, we were able to calculate GSI for 12 individuals. The average GSI was 0.001±0.0008 (mean±s.d.). GSI did not differ between mirror-exposed and control-exposed fish nor between fish exposed to a mirror for 1, 2 or 3 weeks. GSI did not significantly predict gill flaring, latency to gill flare or latency to bite. Interestingly, a linear model showed that GSI demonstrated a non-significant positive trend with cortisol level (*P*=0.058, *F*_1,10_=4.60, *R*^2^=0.25). We therefore conducted a *post hoc* generalized linear model with family instead set to Gamma found a significant positive relationship between GSI and cortisol level (*P*=0.011, χ^2^=1.80, pseudo-*R*^2^=0.46; Fig. 7C).

## DISCUSSION

The present pair of experiments addressed whether behavior, neural response, neurogenesis and physiology are altered by repeated competition in male fighting fish *Betta splendens*, a species artificially selected for territorial behavior. In our study, *B. splendens* maintained metabolically costly territorial aggression daily for 3 weeks. We hypothesize that the maintained aggression observed in our study is due to the inability of the fish to resolve fights with their own mirror image. It is also possible that the costs accrued during our simulated competitions were not high enough to influence subsequent behavior as they only occurred for a short time period once per day. In this study, contests lasted for 10 or 15 min, whereas the metabolic costs of competition for *B. splendens* were established for 30 min fights (Castro et al., 2006; Haller, 1991). However, *B. splendens* do experience some muscle metabolic changes after only 10 min of fighting (Haller, 1991). Peeke and Peeke (1970) argue that short (15 min) periods of stimulation repeated once daily are more naturalistic, given that in the wild a losing fish would typically have the option to flee the encounter. Peeke and Peeke (1970) conducted a study where one cohort engaged in competition for 1 h over 5 consecutive days, whereas another cohort engaged in competition for 15 min over 20 consecutive days (thus both cohorts received 5 h of total competition). In this scenario, both the 15 min and 1 h exposure groups habituated to the exposure by their cumulative 5 h, though the rates at which specific behaviors showed consistent habituation varied across the two groups.

While gill flaring is a stereotyped and frequent aggressive behavior, future studies should assess other displays such as tail beating (a rapid bending of the body in an ‘S’ formation) and fin spreading/tail flashing (a fan-like spreading of the dorsal, ventral and caudal fins) (Dupeyron and Wallace, 2023; Evans, 1985; Simpson, 1968). When in direct competition with another individual (in comparison to our mirror exposure), competing fish may face their opponent head-on, swim parallel to their opponent in a ‘lateral display’ and ‘carousel’ by circling around each other (Simpson, 1968). While prior studies have not reported differences in gill flaring between a mirror and a live competitor (Arnott et al., 2016; Ramos et al., 2021; Crescencio et al., 2024), true competition with another competitor may induce additional behavioral differences not described here (Goldstein, 1975).

Also of note, in addition to engaging in territorial behavior, male *B. splendens* have sole responsibility for parental care of eggs and larval fish (Jaroensutasinee and Jaroensutansinee, 2000; Portugal, 2023). Prior to mating, males construct bubble nests on the surface of the water to ensure eggs receive sufficient oxygen despite the oxygen-poor environments in which they reside (Jaroensutasinee and Jaroensutansinee, 2000; Kang and Lee, 2010). Therefore, it is feasible that parental care behaviors could be influenced by repeated competition, but these were not addressed in the present study.

While hormones have been investigated in *B. splendens* as previously described, the role of the brain is relatively underexplored (but see Clotfelter et al., 2010; Dupeyron and Wallace, 2023; Everett et al., 2025). In our study, the Vs exhibited increased neural activation during social competition in *B. splendens* but, like gill flaring, did not wane over 3 weeks. The Vs is the putative homolog of the mammalian extended amygdala, which includes the medial amygdala and bed nucleus of the stria terminalis (O'Connell and Hofmann, 2011; Magalhães Horn and Rasia-Filho, 2018; Folgueira et al., 2004). Stimulation of the Vs increases aggression in bluegill fish (Demski and Knigge, 1971), spawning in sockeye salmon (Satou et al., 1984) and courtship color change in bluehead wrasse (Luong et al., 2023). Importantly, the extended amygdala is one of two brain regions that exhibits bilateral projection to both social behavior and mesolimbic reward pathways (O'Connell and Hofmann, 2011). In cichlids (*Astatotilapia burtoni*), the Vs neural response differs across social exposures (Rodriguez-Santiago et al., 2022; Jackson et al., 2025). In New Zealand spotty wrasse, Vs pS6 expression is highest in fish attaining dominance following social disruption (Quertermous et al., 2025), reflecting the same directionality as our results.

We further observed a positive relationship between the Vs neural response and the amount of gill flaring displayed over the final 3 days of the experiment. We believe that given there is highly repeatable individual variation in gill flaring, averaging over the final 3 days assesses consistent behavioral tendencies. Few studies have reported relationships between neural response and individual social behavior in fish, though a study in guppies found a positive correlation between social behavior and pS6 expression in the lateral nucleus of the ventral telencephalon (Vl), a putative homolog of the lateral septum (Fischer et al., 2018), and another found that submission behavior positively correlated with *egr-1* expression, a marker of neural activity, in the telencephalon in *Neolamprologus pulcher* (Kasper et al., 2018).

We observed no impact of repeated competition on two separate measurements of stress: behavior in a scototaxis assay and baseline waterborne cortisol. Our baseline cortisol measurements were not intended to capture the typical patterns observed post-fight (Earley et al., 2006). Our results support the conclusion that any acute stress effects of competition do not cause chronic deficits in domesticated *B. splendens*. Ramos et al. (2021) similarly found no association between cortisol and individual aggressive behavior, and Dupeyron and Wallace (2023) found that aggression was not correlated to scototaxis behavior. Further pointing to the independence of behavioral and physiological stress responses in this species, Tudor et al. (2019) observed that the increase in anxiety-like behavior in female *B. splendens* after acute exposure to arsenic did not lead to an increase in cortisol. We hypothesize this is indeed related to the domestication process, as exposure to an unfamiliar environment has been shown to raise cortisol in wild but not domesticated *B. splendens* (Verbeek et al., 2007).

Lastly, it is not surprising that GSI and cortisol exhibited a positive relationship in our study. In trout (*Salmo trutta caspius*), GSI positively correlates with cortisol (Jamalzadeh et al., 2013). More broadly, during spawning, plasma cortisol is elevated in multiple fish species (Delahunty et al., 1979; Maule et al., 1996; Semenkova et al., 2002). Future research should continue to investigate the endocrine mechanisms of reproduction in *B. splendens*. For example, it is well described that higher levels of circulating androgens are correlated with increased aggression in many species (Allee et al., 1955; Cardwell and Liley, 1991; Higby et al., 1991; Hsu et al., 2006), and glucocorticoids can suppress androgen production (Consten et al., 2001a,b; Pankhurst and Van Der Kraak, 2000).

To conclude, we rationalize that *B. splendens* are able to maintain territorial aggressive displays, and the recruitment of the neural mechanisms of social decision making needed to coordinate these displays, without evidence of chronic stress because of their unique domestication history. Domestication in other species has been shown to pleiotropically influence a variety of traits including stress responses (Milla et al., 2021) and brain morphology (Katajamaa and Jensen, 2020). Many species have undergone domestication for morphological traits, but humans have more rarely orchestrated domestication for social traits such as docility in wolves (and silver foxes, see Dugatkin, 2018). In *B. splendens*, we have a noteworthy model to study the consequences of a domestication process that has increased, rather than decreased, aggressive tendencies.

## Supplementary Material

10.1242/jexbio.252766_sup1 Supplementary information
