# Supplementary material for: Repeated competition influences neural response, but not neurogenesis or physiology, in the fighting fish Betta splendens
Source: J Exp Biol. 2026 Oct 6;229(19):jeb252766. doi: 10.1242/jeb.252766 (PMC13723399; doi:10.1242/jeb.252766)
Supplement: Supplementary file 1 [file jexbio-229-252766-s1.pdf]

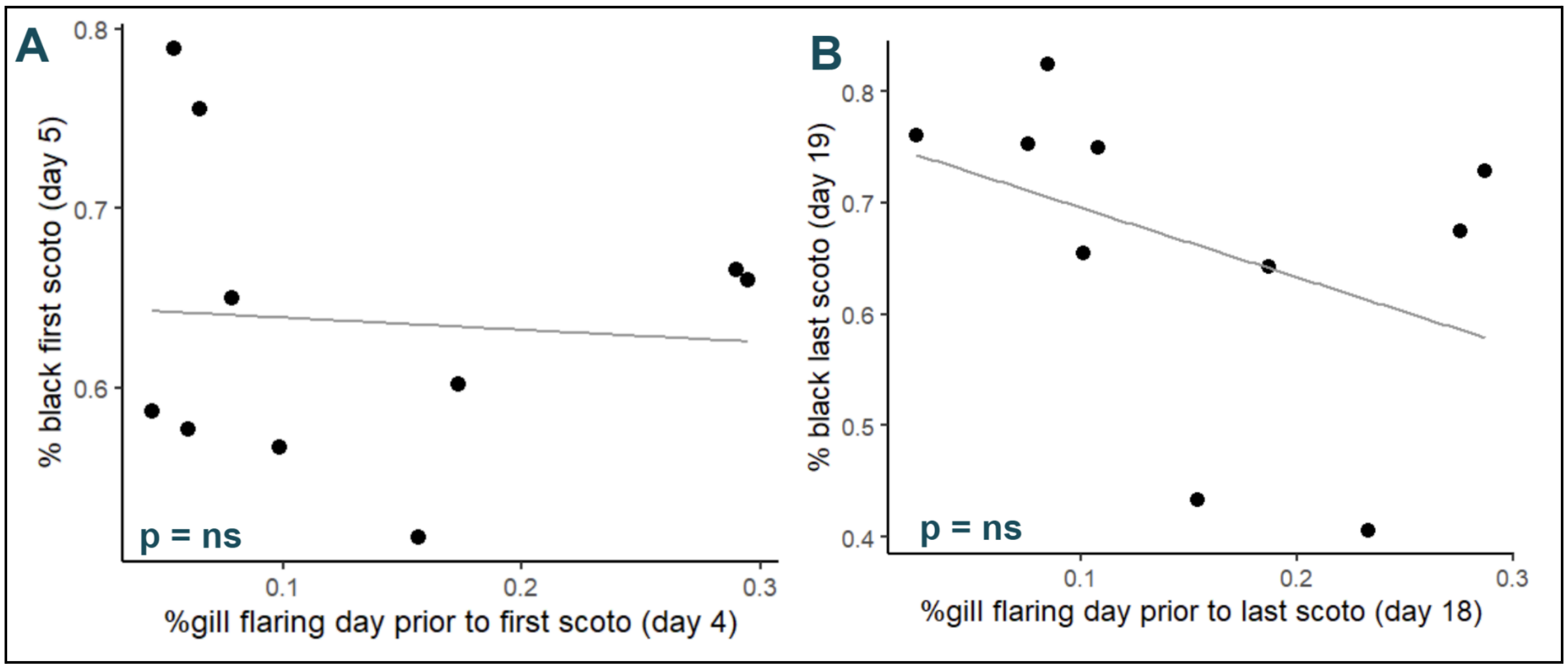

Fig. S1. In experiment 1, anxiety levels (quantified by behavior in a scototaxis assay) did not correlate to aggressive behavior in a mirror exposure on the following day.

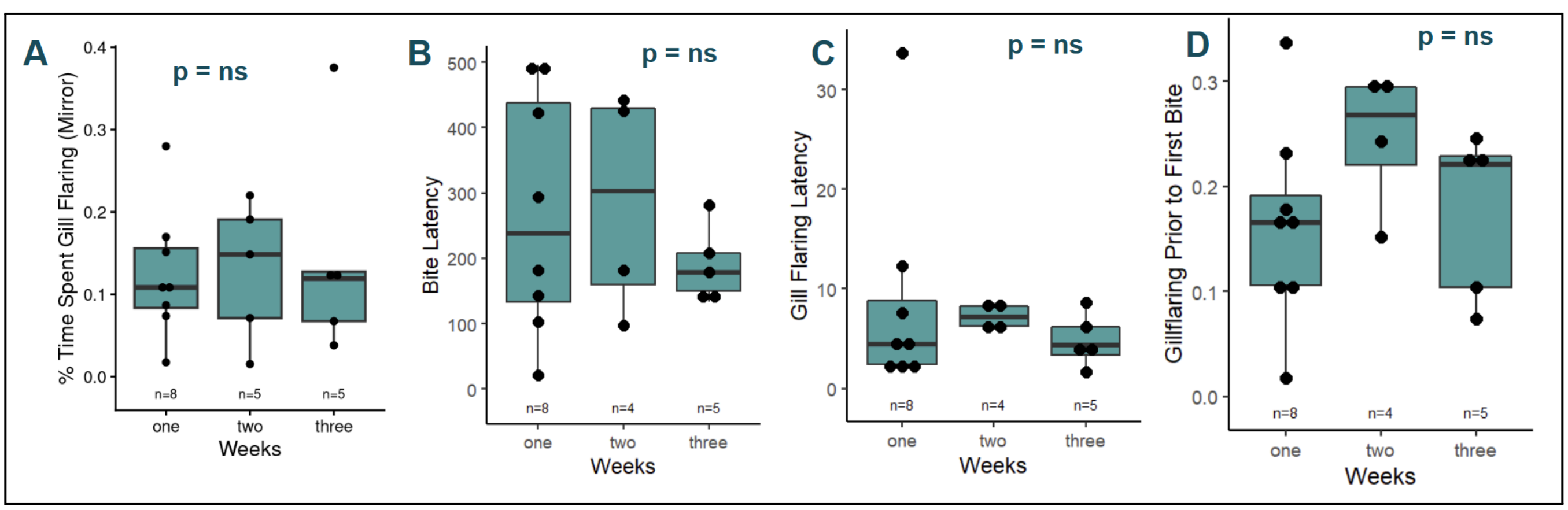

Fig. S2. In experiment 2, measures of aggression (%time spent gill flaring (A) bite latency(B), gill flaring latency(C), and proportion of gill flaring prior ot first bite (D)) did not differ between fish exposed to competition for different lengths of time.

Journal of Experimental Biology • Supplementary information

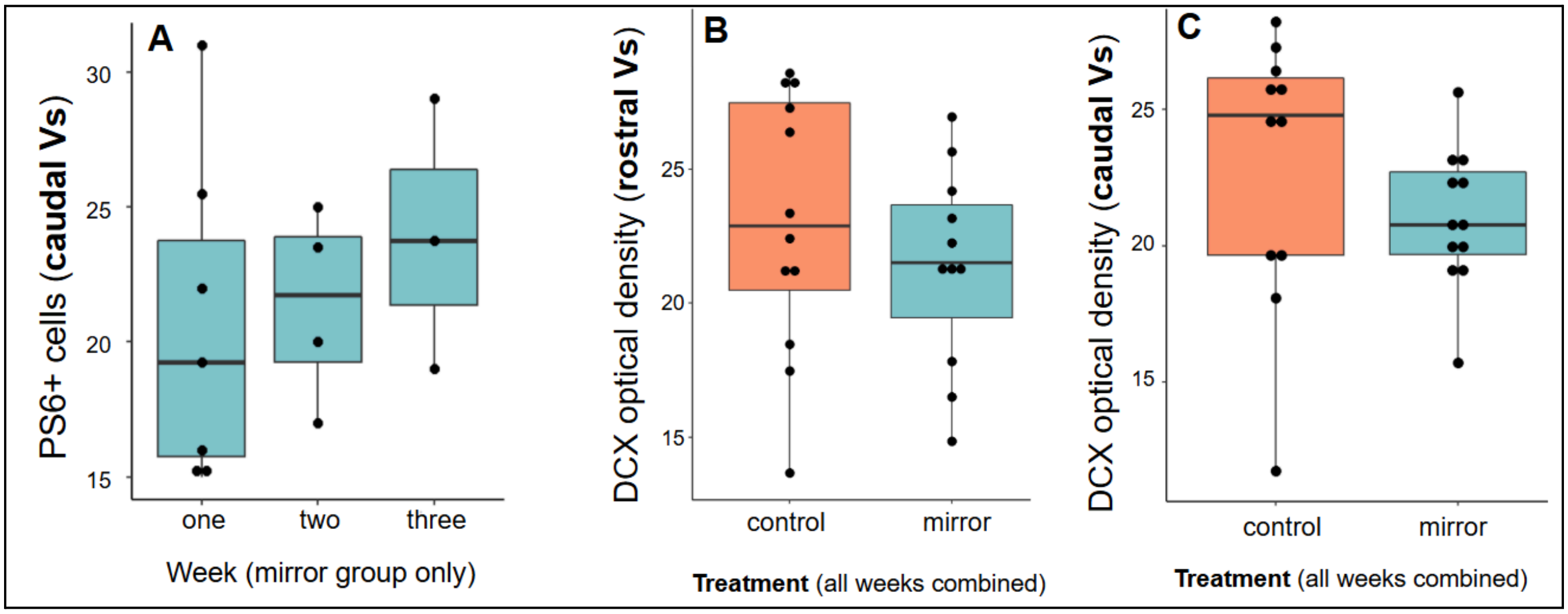

Fig. S3. In experiment 2, Vs PS6 did not differ across length of competition for fish exposed to their mirror image (A). Neurogenesis as measured by doublecortin (DCX) in the rostral (B) and caudal (C) Vs did not differ between fish that were repeatedly exposed to a social competition (mirror stimulus) versus control stimulus.

Table S1. "Treatment" refers to mirror versus control exposure. Bolded values in grey boxes indicate p < 0.05. Note that the supplemental table rows are presented in rough order of their appearance in the main text, and in direct order of their appearance in the data analysis R code.

independent variable	dependent variable	p-value	effect size	statistical test used
Experiment 1				
treatment	% gill flaring (day 1)	**0.002**	1.61	t-test
treatment	% gill flaring (day 18)	**<0.001**	2.26	wilcoxon rank sum test
day 1 vs 18 (control only)	% gill flaring	**0.016**	1.07	paired wilcoxon rank sum test
day 1 vs 18 (mirror only)	% gill flaring	0.106	0.37	paired wilcoxon rank sum test
treatment	scototaxis % black (day 5)	0.433	0.36	t-test
treatment	scototaxis % black (day 19)	0.129	0.71	t-test
day 5 vs 19 (mirror only)	scototaxis % black	0.477	-0.22	paired t-test
day 5 vs 19 (control only)	scototaxis % black	0.523	0.28	paired t-test
scototaxis % black day 5	scototaxis % black day 19	0.069	0.13	linear regression
gill flaring rank (day 1)	gill flaring rank (day 18)	**0.027**	0.48	rank-based linear regression
gill flaring (day 4)	scototaxis % black day 5	0.836	-0.12	linear regression
gill flaring (day 18)	scototaxis % black day 19	0.249	0.06	linear regression
Both Experiments				
gill flaring average experiment 1	gill flaring average experiment 2	0.212	0.49	t-test
Experiment 2 (Behavior & Physiology)				
gill flaring average (original week 1 cohort)	gill flaring average (late-added week 1 cohort)	0.398	-0.67	t-test
treatment	% time near stimulus	**<0.001**	-2.12	wilcoxon rank sum test

treatment (week 1 only)	% time near stimulus	**0.029**	-1.71	wilcoxon rank sum test
treatment (week 2 only)	% time near stimulus	**0.016**	-2.74	wilcoxon rank sum test
treatment (week 3 only)	% time near stimulus	0.095	-1.88	wilcoxon rank sum test
50%	% time near stimulus (control only)	**0.031**	-0.62	wilcoxon signed rank exact test
gill flaring prior to first bite (mirror only)	bite latency (mirror only)	0.705	0.01	generalized linear model
gill flaring day 1	gill flaring day 2	**<0.001**	0.67	linear regression
gill flaring day 1 (mirror only)	gill flaring day 3 (mirror only)	**<0.001**	0.67	linear regression
subject	gill flaring (final 3 days)	–	0.83 (icc)	intraclass correlation coefficient
week (mirror only)	% time near stimulus	0.982	0	kruskal-wallis test
week (control only)	% time near stimulus	0.865	0	kruskal-wallis test
week (mirror only)	% gill flaring	0.930	0.009	anova
week (mirror only)	bite latency	0.600	0.07	anova
week (mirror only)	gill flaring latency	0.352	0.01	kruskal-wallis test
week (mirror only)	gill flaring prior to first bite	0.296	0.16	anova
week	gsi	0.55	0.26	anova
treatment	gsi	0.316	0.05	anova
gsi	% gill flaring	0.388	-0.02	generalized linear model
gsi	gill flaring latency	0.849	0.00	generalized linear model
gsi	bite latency	0.168	0.18	generalized linear model
gsi	cortisol	0.058	0.25	linear regression
gsi	cortisol	**0.011**	0.46	generalized linear model
week (mirror only)	cortisol	0.373	0.00	kruskal-wallis test
treatment	cortisol (all weeks combined)	0.636	0.19	wilcoxon rank sum exact test
% gill flaring (day 1)	cortisol (all weeks combined)	0.575	-0.04	linear regression

Table S2. "Treatment" refers to mirror versus control exposure. Vs section is listed first because we report additional post hoc tests not performed in other regions. Bolded values in grey boxes indicate $p < 0.05$.

independent variable	dependent variable	p-value	effect size	statistical test used
Rostral Vs				
treatment	# ps6+ cells	1	-0.55	t-test
week (mirror only)	# ps6+ cells	0.730	0.06	anova
week (control only)	# ps6+ cells	0.359	0.19	anova
% gill flaring (final day)	# ps6+ cells	0.333	0.00	linear regression
gsi	# ps6+ cells	0.298	0.01	linear regression
standard length	# ps6+ cells	0.392	-0.01	linear regression
week (mirror only)	dcx optical density	0.475	0.17	anova
treatment	dcx optical density	1	0.38	t-test (fdr corrected shown)
Caudal Vs				
treatment	# ps6+ cells	**0.041**	-1.18	t-test (fdr corrected shown)
week (mirror only)	# ps6+ cells	0.667	0.70	anova
week (control only)	# ps6+ cells	0.364	0.20	anova
% gill flaring (final day only)	# ps6+ cells	0.147	0.10	linear regression
% gill flaring (3 day average)	# ps6+ cells	**0.015**	0.35	generalized linear model
gsi	# ps6+ cells	0.379	-0.01	linear regression
standard length	# ps6+ cells	0.548	-0.03	linear regression
treatment	dapi optical density	0.44	-0.32	t-test
week (mirror only)	dcx optical density	0.322	0.22	anova
treatment	dcx optical density	1	0.48	t-test (fdr corrected shown)
Total Vs				
treatment	# ps6+ cells	0.533	-0.71	t-test
week (mirror only)	# ps6+ cells	0.949	0.01	anova
week (control only)	# ps6+ cells	0.415	0.16	anova
% gill flaring (final day)	# ps6+ cells	0.239	0.04	linear regression
gsi	# ps6+ cells	0.131	0.10	linear regression
standard length	# ps6+ cells	0.261	0.01	linear regression
Rostral Vd				
treatment	# ps6+ cells	1	0.54	t-test
week (mirror only)	# ps6+ cells	0.720	0.64	anova
week (control only)	# ps6+ cells	0.244	0.27	anova
% gill flaring (final day)	# ps6+ cells	0.384	-0.02	linear regression

gsi	# ps6+ cells	0.326	0.00	linear regression
standard length	# ps6+ cells	0.406	-0.01	linear regression
Caudal Vd				
treatment	# ps6+ cells	0.99	0.62	t-test
week (mirror only)	# ps6+ cells	0.227	0.26	anova
week (control only)	# ps6+ cells	0.949	0.01	anova
% gill flaring (final day)	# ps6+ cells	0.527	-0.05	linear regression
gsi	# ps6+ cells	0.679	-0.08	linear regression
standard length	# ps6+ cells	0.295	0.15	linear regression<br>(fdr corrected shown)
Total Vd				
treatment	# ps6+ cells	1	0.55	t-test
week (mirror only)	# ps6+ cells	0.811	0.03	anova
week (control only)	# ps6+ cells	0.462	0.16	anova
% gill flaring (final day)	# ps6+ cells	0.400	-0.02	linear regression
gsi	# ps6+ cells	0.869	-0.07	linear regression
standard length	# ps6+ cells	0.074	0.10	linear regression
Rostral VvM				
treatment	# ps6+ cells	1	-0.26	t-test
week (mirror only)	# ps6+ cells	0.392	0.17	anova
week (control only)	# ps6+ cells	0.244	0.27	anova
% gill flaring (final day)	# ps6+ cells	0.253	0.04	linear regression
gsi	# ps6+ cells	0.124	0.12	linear regression
standard length	# ps6+ cells	0.356	-0.01	linear regression
Caudal VvM				
treatment	# ps6+ cells	1	0.22	t-test
week (mirror only)	# ps6+ cells	0.304	0.21	anova
week (control only)	# ps6+ cells	0.622	0.11	anova
% gill flaring (final day)	# ps6+ cells	0.923	-0.09	linear regression
gsi	# ps6+ cells	0.375	-0.01	linear regression
standard length	# ps6+ cells	0.490	-0.02	linear regression
Total VvM				
treatment	# ps6+ cells	1	-0.08	t-test

week (mirror only)	# ps6+ cells	0.417	0.15	anova
week (control only)	# ps6+ cells	0.402	0.18	anova
% gill flaring (final day)	# ps6+ cells	0.440	-0.03	linear regression
gsi	# ps6+ cells	0.174	0.08	linear regression
standard length	# ps6+ cells	0.301	0.01	linear regression
Rostral VvL				
treatment	# ps6+ cells	1	0.25	t-test
week (mirror only)	# ps6+ cells	0.266	0.23	anova
week (control only)	# ps6+ cells	0.393	0.19	anova
% gill flaring (final day)	# ps6+ cells	0.342	-0.00	linear regression
gsi	# ps6+ cells	0.105	0.14	linear regression
standard length	# ps6+ cells	0.052	0.14	linear regression
Caudal VvL				
treatment	# ps6+ cells	1	0.49	t-test
week (mirror only)	# ps6+ cells	0.441	0.15	anova
week (control only)	# ps6+ cells	0.984	0.00	anova
% gill flaring (final day)	# ps6+ cells	0.688	-0.07	linear regression
gsi	# ps6+ cells	0.804	-0.09	linear regression
standard length	# ps6+ cells	0.309	0.15	linear regression<br>(fdr corrected shown)
Total VvL				
treatment	# ps6+ cells	1	0.39	t-test
week (mirror only)	# ps6+ cells	0.133	0.31	anova
week (control only)	# ps6+ cells	0.692	0.08	anova
% gill flaring (final day)	# ps6+ cells	0.437	-0.03	linear regression
gsi	# ps6+ cells	0.385	-0.01	linear regression
standard length	# ps6+ cells	0.141	0.19	linear regression
Principal Component Analysis of All Regions				
treatment	PC1	0.931	0.04	t-test
treatment	PC2	**0.008**	-1.26	t-test

Journal of Experimental Biology • Supplementary information
